# Effectiveness of a social cognition remediation intervention for patients with schizophrenia: a randomized-controlled study

**DOI:** 10.1192/j.eurpsy.2025.2182

**Published:** 2025-08-26

**Authors:** P. Pezzella, L. Giuliani, A. Mucci, D. Palumbo, E. Caporusso, G. Piegari, G. M. Giordano, P. Blasio, S. Torriero, C. Mencacci, S. Galderisi

**Affiliations:** 1 University of Campania “Luigi Vanvitelli”, Naples; 2 Department of Psychiatry and Addiction, ASST Fatebenefratelli-Sacco , Milan, Italy

## Abstract

**Introduction:**

Individuals with schizophrenia often experience significant deficits in social cognition, including emotion processing, social perception, and theory of mind (ToM). These deficits have a greater impact than symptoms on occupational and social functioning.

**Objectives:**

The present randomized controlled trial aimed to evaluate the effectiveness of a new integrated and personalized social cognition rehabilitation intervention, the Social Cognition Individualized Activity Lab (SoCIAL), in improving performance in social cognition and consequently the clinical and functional outcome of subjects with schizophrenia (SCZ).

**Methods:**

SoCIAL, consisting of 10 weekly sessions, was compared with treatment as usual (TAU). Two recruitment centers (Naples and Milan) were involved. Repeated measures MANOVA was used to investigate between-group differences in changes from baseline to follow-up in terms of psychopathology, cognitive performance, and functioning.

**Results:**

Twenty people with schizophrenia were blindly assigned to SoCIAL and twenty to TAU. After 10 weeks, SoCIAL significantly improved disorganization, emotion recognition, functional capacity and real-life functioning. As compared to TAU, the SoCIAL group showed a significant improvement in minimal and enriched social inference domain of theory of mind, and in key domains of real-life functioning (interpersonal relationships, everyday life skills, and work skills).

**Conclusions:**

SoCIAL improved social cognition and real-life functioning of people with schizophrenia. These results highlight the importance of social cognition deficit treatment in schizophrenia and the necessity for these interventions to be multifaced and personalized. Such an approach ensures that improvements in social cognition translate into enhanced functional outcomes.

**Disclosure of Interest:**

None Declared

